# Prenatal hypoxia plus postnatal high‐fat diet exacerbated vascular dysfunction via up‐regulated vascular Cav1.2 channels in offspring rats

**DOI:** 10.1111/jcmm.14020

**Published:** 2018-12-16

**Authors:** Xiang Li, Xueqin Feng, Likui Lu, Axin He, Bailin Liu, Yingying Zhang, Ruixiu Shi, Yanping Liu, Xueyi Chen, Miao Sun, Zhice Xu

**Affiliations:** ^1^ Institute for Fetology First Hospital of Soochow University Suzhou China; ^2^ Center for Perinatal Biology Loma Linda University Loma Linda California

**Keywords:** high fat, ion channels, L‐type calcium channels, prenatal, voltage‐gated potassium channels

## Abstract

**Background:**

This study aimed to examine whether and how postnatal high‐fat diet had additional impact on promoting vascular dysfunction in the offspring exposed to prenatal hypoxia.

**Methods and Results:**

Pregnant Sprague‐Dawley rats were randomly assigned to hypoxia (10.5% oxygen) or normoxia (21% O_2_) groups from gestation days 5‐21. A subset of male offspring was placed on a high‐fat diet (HF, 45% fat) from 4‐16 weeks of age. Prenatal hypoxia induced a decrease in birth weight. In offspring‐fed HF diet, prenatal hypoxia was associated with increased fasting plasma triglyceride, total cholesterol, free fatty acids, and low‐density lipoprotein‐cholesterol. Compared with the other three groups, prenatal hypoxic offspring with high‐fat diet showed a significant increase in blood pressure, phenylephrine‐mediated vasoconstrictions, L‐type voltage‐gated Ca2+ (Cav1.2) channel currents, and elevated mRNA and protein expression of Cav1.2 α1 subunit in mesenteric arteries or myocytes. The large‐conductance Ca2+‐activated K+ (BK) channels currents and the BK channel units (β1, not α‐subunits) were significantly increased in mesenteric arteries or myocytes in HF offspring independent of prenatal hypoxia factor.

**Conclusion:**

The results demonstrated that prenatal hypoxia followed by postnatal HF caused vascular dysfunction through ion channel remodelling in myocytes.

## 1 INTRODUCTION

Cardiovascular disease (CVD) is the leading cause of global mortality. It is well known that CVD is associated with risk factors such as adiposity, smoking, ageing, and hypertension.^1^ Numerous studies have revealed significant associations between compromised prenatal environments and the development of CVD in adult offspring, which are known as foetal origins of adult diseases.^2^, ^3^, ^4^

A common clinical complication during pregnancy is hypoxia in utero*,* arising from high altitude,^5^ preeclampsia,^6^ uteroplacental dysfunction,^7^, and other factors. Impaired oxygen supply to foetuses initiates the centralisation of blood flow to vital organs, such as the heart and brain.^5^, ^8^ Although this adaptation is necessary for critical organs, the decreased peripheral blood flow can impair the development of other tissues and results in intrauterine growth retardation (IUGR). Numerous retrospective and prospective studies have revealed that low birth weight is associated with the development of CVD in later life.^3^, ^4^, ^9^

Unhealthy living habits after birth could exacerbate the impact of adverse exposure in utero.^2^ For example, postnatal high salt intake could exacerbate blood pressure (BP) and dysfunction of ion channels in vascular smooth muscle cells (VSMCs) in the offspring exposed to prenatal hypoxia.^10^ Prenatally programmed vulnerability to CVD could be exacerbated by high‐salt diets in postnatal life.^11^ It is well known that high fat intake plays an important role in the pathogenesis of CVD. Previous studies demonstrated that a combination of prenatal hypoxic insult and postnatal high‐fat diets could increase the susceptibility to cardiovascular dysfunction,^12^ the mechanisms of which are still unknown.

Vascular dysfunction with increased arterial tone is a major contributing factor for hypertension. Mesenteric arteries (MA) are typical peripheral resistance vessels. They play an important role in regulating BP based on functions of VSMCs.^13^ In general, large‐conductance Ca^2+^‐activated K^+^ (BK) channels in VSMCs can be activated by membrane depolarisation and intracellular local Ca^2+^ release. Activation of BK channels increases K^+^ efflux, causing cell membrane hyper‐polarisation, and deactivation of voltage‐dependent Ca^2+^ channels, ultimately resulting in vascular relaxation.^14^ Therefore, the coordination of BK and voltage‐dependent Ca^2+^ channels in VSMCs are critical in regulating vascular tone. However, whether and how those two channels may contribute to mesenteric artery dysfunctions in the offspring exposed to prenatal hypoxia and postnatal high‐fat diet is largely unknown.

The present study established a rat model: following prenatal hypoxia, the offsprings were given either high fat or normal diets during early postnatal life. Then, we assessed the impact of chronic prenatal hypoxia or/and postnatal high fat intake on arterial blood pressure, functional resistance vessels, and ion channels of mesenteric artery smooth muscle cells (MASMCs), including BK and Cav1.2 channels in male young adult offspring. We suggested that vascular or cellular functions, as well as ion channel activities, would be affected by prenatal hypoxia, which would be further exacerbated by postnatal high fat intake during early life. The new information gained may provide a better understanding for the mechanisms of vascular dysfunction caused by prenatal hypoxia and postnatal high‐fat diet, and would help for early prevention and treatments of hypertension in developmental origins.

## 2 MATERIALS AND METHODS

All experimental procedures were approved by the Institutional Animal Care and Use Committee of Soochow University and performed in accordance with the Guide for the Care and Use of Laboratory Animals (National Research Council, 2011).

### 2.1 Animals

Sprague**‐**Dawley rats (Su Pusi Biotech., Suzhou, China), 240‐270 g, were allowed access to standard food and tap water ad libitum and housed under a 12 hours light‐dark cycle. After acclimatisation for a week, female rats were mated and pregnancy was confirmed by the presence of vaginal plug observed the following day, which was designated as gestational day (GD) 0. On GD 5, pregnant rats were randomly assigned to normoxia control (Con) and prenatal hypoxia (PH) group. From GD 5 to GD 20, the control rats were housed in a chamber filled with room air, while the hypoxia group was treated with the same chambers infused with nitrogen to maintain the oxygen concentration at 10.5%. On GD 21, all dams were moved out from chambers for natural delivery.

A subset of male offspring aged 4 weeks (n = 26 offspring from 13 L, each group) was randomly allocated to receive either a high‐fat (HF) diet (45% fat) or a normal diet with low fat (LF) (5% fat, Slacom, Shanghai, China). Then, four groups were created: normoxia control offspring with the LF diet (CLF; n = 13 from 13 L), normoxia control offspring with the HF diet (CHF; n = 13 from 13 L), prenatal hypoxia offspring with the LF diet (HLF; n = 13 from 13 L), and prenatal hypoxia offspring with the HF diet (HHF; n = 13 from 13 L). Those feeding were provided for 12 weeks before testing.

### 2.2 Measurement of blood pressure

Male young adult offspring rats (n = 7‐10/group) were anaesthetized with sodium pentobarbitone (50 mg/kg, IP) for measurement of BP as described.^15^ Polyethylene catheters filled with heparin were implanted in the femoral artery and were tunnelled subcutaneously, externalized at the nape of the neck. Two days after surgical recovery, BP was recorded in conscious, freely moving rats at the same time for all rats (4:00 pm). The baseline BP was monitored for 1 hour using the Power‐Lab system and software (AD Instruments, Bella Vista, NSW, Australia).

### 2.3 Plasma analyses

After an overnight fast from 8:00 pm to 8:00 am, offspring rats were killed using sodium pentobarbital (100 mg/kg, i.p.). Blood samples were collected from the abdominal aorta with heparin sodium. After being centrifuged at 2000 *g* for 10 minutes, plasma was collected for the determination of triglyceride (TG), total cholesterol (TC), low‐density lipoprotein‐cholesterol (LDL‐C), high‐density lipoprotein‐cholesterol (HDL‐C), and free fatty acid (FFA). TG, TC, FFA, LDL‐C, and HDL‐C were determined by an automatic spectrophotometer according to the manufacturer's protocols, the testing kits were purchased from Nanjing Jiancheng Bioengineering Institute.

### 2.4 Measurement of vessel tone

The third order of mesenteric arteries of 4‐month‐old male offspring were excised and cut into small rings (2 mm in length) in Krebs‐Henseleit solution containing (mmol/L) 125 NaCl, 4.6 KCl, 1.2 MgCl_2_, 13.5 NaHCO_3_, 1.2 NaH_2_PO_4_, 2.5 CaCl_2_, 0.025 EDTA, and 10 glucose; pH 7.4 with NaOH. The solution was maintained at 37°C and continuously gassed with 95% O_2_‐5% CO_2_. Each segment was threaded onto two 40‐μm tungsten wires and mounted in a myograph system (610M, Danish Myotechniques, Aarhaus, Denmark).

The mesenteric arteries were given a testing tension of 0.9 of L13.3 kPa by using a normalisation software package^10^ (Myodata, Danish Myotechnologies) and equilibrated for 1 hour in Krebs‐Henseleit solution gassed with 95%O_2_‐5%CO_2_. Vessel viability was assessed first by repeated exposure to 60 mmol/L KCl. After washing and stabilisation, induced vasoconstrictions were obtained by adding cumulative concentrations of phenylephrine (PE, 10^−9^ to 10^−4^ mol/L) to chambers. Acetylcholine (Ach, 10^−9^ to 10^−4^ mol/L) was used following the application of PE (10^−4 ^μmol/L) that produced and maintained steady vasoconstrictions at least 15‐20 minutes. After washing, concentration‐response curves to PE (10^−9^ to 10^−4^ mol/L) were obtained in the presence and absence of Cav1.2 inhibitor nifedipine (Nife, 1 μmol/L, 30 minutes), or BK channel specific blocker iberiotoxin (IbTx, 0.1 μmol/L, 1 hour).

### 2.5 Isolation of SMCs from offspring MA

Single VSMCs were enzymatically dissociated from mesenteric arteries as previously described.^10^ Briefly, arterial segments from the third‐order MA were placed in an ice‐cold Ca^2+^‐free physiological saline solution (PSS) containing (in mmol/L) 137 NaCl, 5.6 KCl, 1 MgCl_2_, 0.44 NaH_2_PO_4_, 0.42 Na_2_HPO_4_, 4.2 NaHCO_3,_ 10 HEPES and 10 glucose (pH 7.4 with NaOH). Vascular pieces were then exposed to a two‐step digestion process: (a) 15 minutes incubation in PSS (37°C) containing 0.5 mg/ml papain (Solarbio, China), 2 mg/mL ABV and 1.5 mg/mL dithioerythritol (BioSHARP, China); and (b) a 5‐12 minutes incubation in PSS (37°C) containing 0.7 mg/mL type F collagenase and 0.4 mg/mL type H collagenase (Sigma, USA). Then tissues were washed repeatedly with ice‐cold Ca^2+^‐free PSS and triturated with a fire‐polished pipette. Liberated cells were then stored in Ca^2+^‐free PSS at 4°C for use within ~6 hours.

### 2.6 Electrophysiology

Conventional whole‐cell configuration was used to monitor whole‐cell K^+^ currents. SMCs were equilibrated in bath solution (in mmol/L): 135 NaCl, 5 KCl, 1 MgCl_2_, 1.8 CaCl_2_, 10 glucose, and 10 HEPES (pH 7.4). Recording electrodes (3‐5 MΩ) were pulled from borosilicate glass micro‐capillary tubes using a horizontal pipette puller (P‐97, Sutter Instrument Co, Novato, CA) and backfilled with internal solution (in mmol/L): 110 potassium aspartate, 0.85 CaCl_2_, 30 KCl, 3 Na_2_ATP, 1 EGTA, 10 HEPES and 10 glucose; pH 7.2 with KOH. To record outward K^+^ currents, isolated cells were held at –70 mV and then exposed to voltage steps ranging from –60 to +60 mV (10 mV intervals, 500 ms). Whole‐cell BK channel currents were defined as 0.1 μmol/L IbTx‐sensitive components obtained by the digital subtraction of traces in the presence of IbTx from baseline traces.

For measurement of whole‐cell Cav1.2 currents, conventional whole‐cell patch‐clamp configuration was conducted and BaCl_2_ (20 mmol/L) was used as a charge carrier to resist current rundown. The bath solution contained (in mmol/L): 20 BaCl_2_, 10 HEPES, 10 glucose, 1 MgCl_2_, and 125 TEA (pH 7.3 with TEA‐OH). The pipette (3‐5 MΩ) solution contained (in mmol/L): 140 caesium glutamate, 10 HEPES, 3 Na_2_ATP, 1 MgCl_2_, 10 Glucose, 10 EGTA, and (pH 7.3 with CsOH). Ba^2+^ current was recorded at a voltage range from –60 to +60 mV with 10 mV increments (300 ms duration) from a holding potential of –70 mV. The whole‐cell recordings used for analysis should meet the following conditions: a series resistance <20 MΩ, leakage current <100pA, and seal resistances >2 GΩ.

BK single‐channel currents from isolated SMC were recorded using the inside‐out patch‐clamp configurations. Pipette (pH 7.2 with KOH) and bath (pH 7.4 with KOH) solutions contained (in mmol/L): 145 KCl, 10 HEPES, 1 EGTA and 5 glucose. Free Ca^2+^ in bath solution was adjusted to the desired concentration by adding CaCl_2_, which was determined by using MaxChelator software (Chris Patton, Stanford University, USA). The number of channels in each patch (N) and the single channel open probability (Po) were used as an index reflecting the channel steady‐state activity. The BK channel activity (NPo) was calculated using the following equation:

NPo=Σ(t1+t2…ti)

where it is the relative open time (time open/total time) for each channel level. Po was calculated by dividing NPo according to the total number of channels per patch. The total number of BK channels in each patch was determined at a voltage of +80 mV with 10 μmol/L Ca^2+^ in the bath solution.^16^ Recordings can be used for analysis when the stable Po values continued for a minimum of 2 minutes.

Voltage‐sensitivity data were fitted with the Boltzmann function:

$$\text{Po}=1/\{1+\text{exp}\left[-\text{ZF/RT}\left(V-V_{1/2}\right)\right]\}$$

where V_1/2_ is the voltage of half‐maximal channel activation. The Ca^2+^‐ sensitivity data were fitted with the Hill equation:

$$\text{Po}={\left[Ca^{2+}\right]}_{i}\eta^{H}/K_{d}\eta^{H}+{\left[Ca^{2+}\right]}_{i}\eta^{H}$$

where η^H^ is the Hill co‐efficient, and K_d_ is the dissociation constant defined as [Ca^2+^]_i_ required for half activation.

All patch‐clamp experiments were performed with Axon700B amplifier and Clampfit 10.1 software (Axon Instruments, Foster City CA) at 23°C. Membrane currents were sampled at 10 kHz and filtered at 2 kHz with a 8 pole Bessel filter and then stored for subsequent analysis using Clampfit 10.1 software. Cell capacitance was measured through the cancellation circuitry of the voltage‐clamp amplifier.

### 2.7 Real‐time quantitative PCR (RT‐qPCR)

Total RNA was extracted from freshly isolated MA with RNAiso Plus Trizol (Takara, Japan). RNA was then reverse transcribed into cDNA with the RevertAid First Strand cDNA Synthesis Kit (Thermo Scientific, USA). The reference primer sequences for qPCR assays were acquired from previous the study.^17^, ^18^ The qPCR was performed with a SYBR^®^ Premix Ex Taq™ mix (TaKaRa, Japan). Data were normalized against β‐actin as internal control and calibrated with a normal control cDNA. The relative expression ratio was calculated with the 2^−ΔΔCt^ method.

### 2.8 Western blotting

Mesenteric arteries were homogenized in lysis buffer containing a cocktail of protease inhibitors. After incubation on ice for 30 minutes, the homogenate was centrifuged at 13 800 *g* (30 min, 4°C). Then the supernatant was collected and protein concentration was measured using the Bradford protein assay. Equal amount of protein (50 μg) from each group was loaded for gel electrophoresis and then electrophoretically transferred to polyvinylidene fluoride membrane. After blocked with 5% bovine serum albumin prepared in Tris‐buffered saline containing 0.2% Tween‐20 (TBST), the membranes were incubated with different subunit‐specific primary antibodies overnight at 4°C. The antibodies included specific polyclonal antibodies (Cav1.2α1C, 1:200; BKα, 1:500; BK β1,1:500; Alomone, Jerusalem, Israel; β‐actin,1:2,000; Beyotime Biotech, Shanghai, China). After three washes with TBST, the membrane was incubated with secondary horseradish peroxidase‐conjugated goat anti‐rabbit antibody (1: 4000) for 2 hours at room temperature. The immunoreactive bands were identified using enhanced chemiluminescence, and signals were recorded using an Imaging System (Tanon, Shanghai). Protein bands were quantified using Quantity One software (Bio‐Rad).

### 2.9 Statistical analysis

All data are presented as the mean ± SEM and analysed using GraphPad Prism, version 5.0 (GraphPad Software, San Diego CA). Data were analysed with one‐way or two‐way anova followed by Bonferroni post hoc tests. *P* < 0.05 was considered statistically significant.

## 3 RESULTS

### 3.1 Body weight and food intake

**Figure 1:**
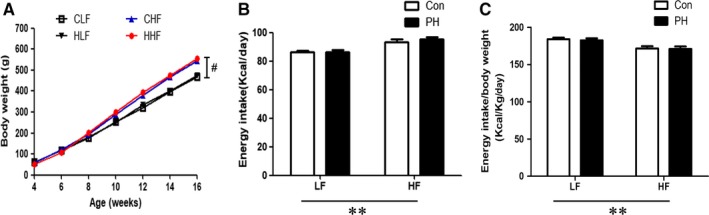
The effect of prenatal hypoxia and postnatal high‐fat (HF) diets on body weight (A), energy intake (B), and absolute energy intake adjusted by body weight (C). LF: Low fat (n = 13 by group). CLF, control offspring with the LF diet; CHF, control offspring with the HF diet; HLF, prenatal hypoxia offspring with the LF diet; HHF, prenatal hypoxia offspring with the HF diet; PH, prenatal hypoxia.**P < 0.001, two‐way anova. #*P* < 0.05, Bonferroni post hoc test, comparing between the PH and control offspring receiving the same diet

Prenatal hypoxia caused a significant decrease in body weight at birth (Con: 6.39 ± 0.09 g vs PH: 5.79 ± 0.07 g, *P* < 0.001, n = 13). However, at 4 weeks of age, there was no difference in the body weight between PH and control pups (Figure 1A). After 12 weeks of feeding, the HF‐fed offspring gained more weight than that of LF offspring (Figure 1A). Compared with the offspring on LF diet, in HF‐fed offspring, the ratio of energy intake to body weight was decreased, while the total energy intake per animal was significantly increased (Figure 1B, C).

### 3.2 Effect of prenatal hypoxia and postnatal HF diet on lipid profiles and blood pressure

High‐fat diet increased plasma lipid concentrations, including TG, TC, FFA, and LDL‐C, and decreased HDL‐C in both control and PH‐exposed rats (Table 1). PH‐exposed and HF‐fed rats exhibited higher concentrations of TG, TC, LDL‐C, and FFA than HF‐fed control offspring, and an interaction of PH and HF in elevated plasma FFA was observed (Table 1). Besides, PH alone also increased plasma TC, LDL‐C, and FFA compared to the control (Table 1).

**Table 1:** Lipid profiles, blood pressure, and heart rate in a 4‐month‐old male offspring

	LF diet		HF diet		Two‐way anova		
	Con	PH	Con	PH	PH	Diet	Int
TG (mmol/L)	0.51 ± 0.03	0.53 ± 0.07	0.71 ± 0.04	0.89 ± 0.03†	*	*	
TC (mmol/L)	1.43 ± 0.12	2.03 ± 0.18	2.24 ± 0.18	2.52 ± 0.25	*	*	
HDL‐C (mmol/L)	1.51 ± 0.10	1.32 ± 0.11	1.08 ± 0.06	1.04 ± 0.08		*	
LDL‐C (mmol/L)	0.30 ± 0.04	0.53 ± 0.05†	0.96 ± 0.07	1.39 ± 0.08†	*	*	
FFA (mmol/L)	0.47 ± 0.01	0.48 ± 0.01	0.65 ± 0.02	0.74 ± 0.02†	*	*	*
SBP (mm Hg)	122.20 ± 0.8	119.10 ± 3.0	137.06 ± 1.9	144.78 ± 3.2†		*	*
DBP (mm Hg)	75.3 ± 2.8	83.7 ± 6.1	84.4 ± 1.4	93.0 ± 2.8	*	*	
HR (mm Hg)	324.8 ± 2.6	320.7 ± 10.4	351.7 ± 7.2	356.6 ± 10.6		*	

Con, control; HF, high fat; Int, interaction; LF, low fat; PH, prenatal hypoxia.

* *P* < 0.05 for the respective sources of variation (PH, diet, or their interaction) using two‐way anova (Lipid profiles: n = 6 per group; Blood pressure and heart rate: Con and PH with LF diet, n = 7; Con with HF diet, n = 9; PH with HF diet, n = 8).

† *P* < 0.05 (Bonferroni post hoc test compared to the control offspring fed with the same diet).

After 12 weeks of the feeding, the baseline blood pressure, including SBP and DBP were increased in HF‐fed rats (Table 1). With LF diet, there was no difference in SBP and DBP between the PH‐exposed and control rats. However, with HF diet, SBP and DBP were significantly increased in PH‐exposed rats compared to the control (Table 1). Heart rate was increased in HF‐fed rats independently from prenatal hypoxia factor (Table 1).

### 3.3 Effect of prenatal hypoxia and postnatal HF diet on vasoconstriction and vasodilatation

**Figure 2:**
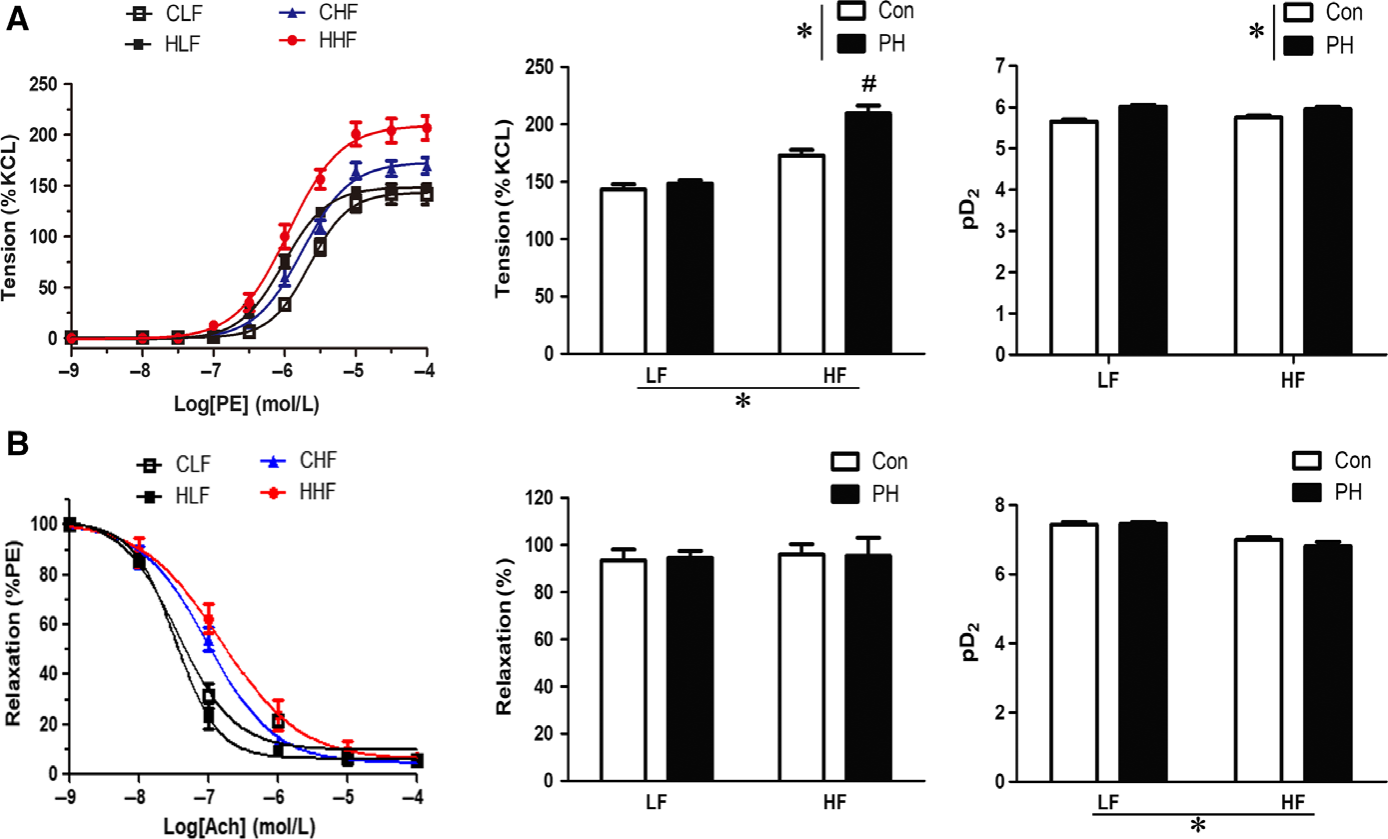
The effect of prenatal hypoxia and postnatal HF diet on phenylephrine (PE)‐induced vasoconstriction and ACh‐mediated vasodilatation in offspring mesenteric arteries (MA). A, PE‐increased dose‐response contractions (n = 7 from 7 mothers each group). B, Vasodilatation in the MA (n = 6 from 6 mothers each group). Data in A were expressed as a percentage of the maximal contraction induced by 60 mmol/L KCL (%Kmax). The relaxation responses to acetylcholine (Ach) in B were expressed as a percentage of the PE‐induced contraction. The vascular sensitivity was expressed as pD2 (–log EC50). CLF, control offspring with the LF diet; CHF, control offspring with the HF diet; HLF, prenatal hypoxia offspring with the LF diet; HHF, prenatal hypoxia offspring with the HF diet; PH, prenatal hypoxia. *P < 0.05, two‐way anova. #*P* < 0.05, Bonferroni post hoc test, comparing between the PH and control offspring receiving the same diet

In ex vivo vessel ring contraction experiment, there was no difference in KCl‐induced contraction (60 mmol/L) in all groups (CLF = 5.90 ± 0.38 mN, n = 12 vessel rings from seven animals; HLF = 6.12 ± 0.58 mN, n = 13 vessel rings from seven animals; CHF = 6.35 ± 0.62 mN, n = 14 vessel rings from seven animals; HHF = 6.60 ± 0.58 mN, n = 13 vessel rings from seven animals), while the maximal response of PE‐induced vasoconstrictions was significantly higher in mesenteric arteries of HF‐fed offspring (Figure 2A). Prenatal hypoxia resulted in a further significant increase of PE‐induced vasoconstrictions compared with the control group (Figure 2A). Although prenatal hypoxia alone did not change the maximal response of PE‐induced vasoconstrictions, the pD_2_ (–logEC50) values were significantly increased (Figure 2A). Furthermore, the pD2 (–logEC50) values in ACh‐mediated dose‐dependent vasodilatation were significantly decreased in HF‐fed offspring independent of the prenatal hypoxia, while the maximal relaxation was not changed among the four groups (Figure 2B).

### 3.4 Effect of prenatal hypoxia and postnatal HF diet on BK functions and protein expression

**Figure 3:**
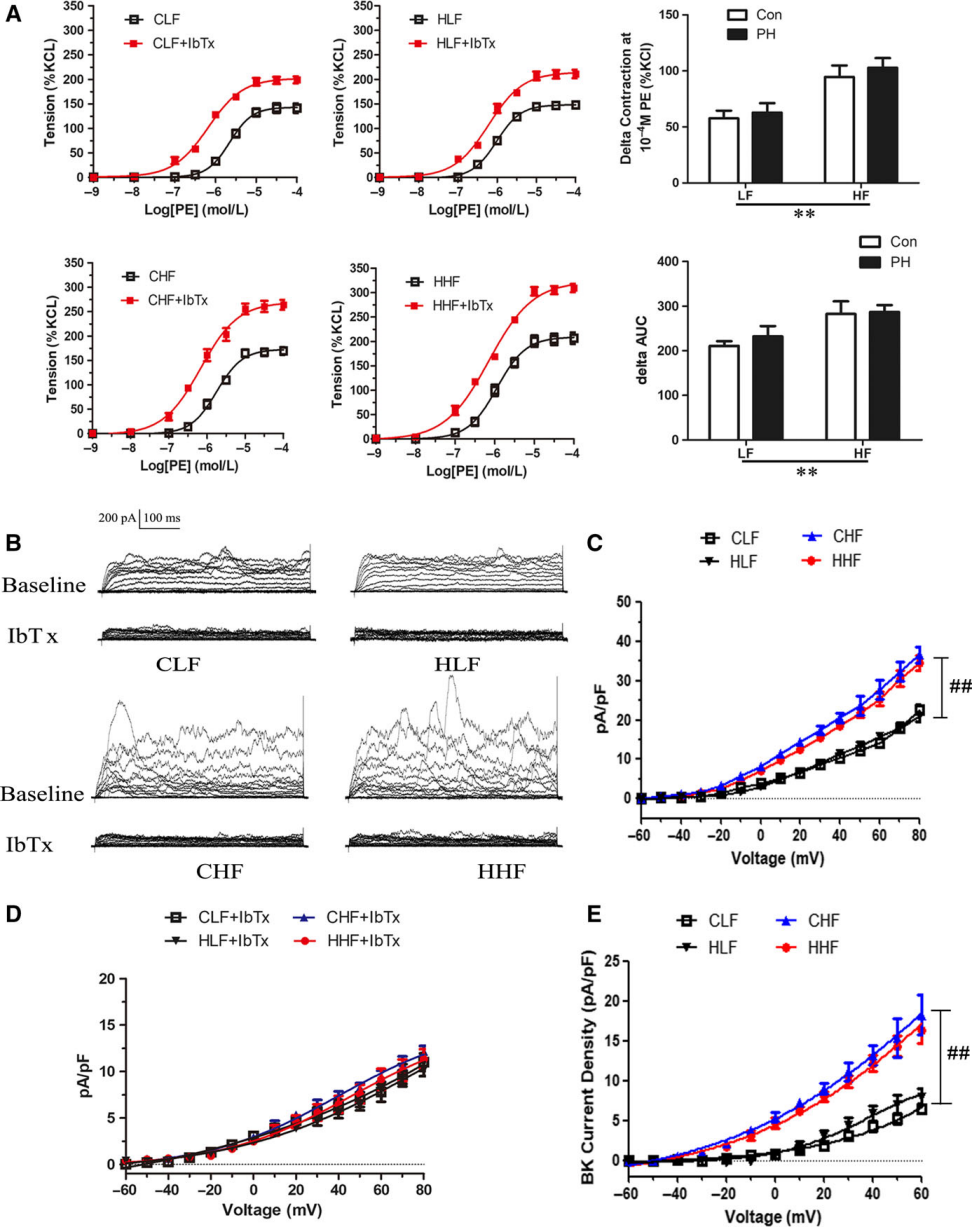
The effect of prenatal hypoxia and postnatal HF diets on large‐conductance calcium‐activated K^+^ (BK) channels in mesenteric arteries and whole‐cell K^+^ current density in mesenteric artery myocytes. A, The effect of iberiotoxin (IbTx) on PE‐induced contraction in MAs from the offspring of CLF, HLF, CHF and HHF. B, The maximal increase of PE‐induced vessel contraction (ΔPE) after IbTx pre‐treatment. C, Representative recordings of whole‐cell K^+^ currents measured during depolarising voltage steps in mesenteric artery myocytes. I‐V relationships of mean whole‐cell K^+^ current density in absence (D) or presence (E) of IbTx in myocytes. F, I‐V relationships of iberiotoxin‐sensitive BK current density in myocytes. AUC, area under the concentration‐response curve; CLF, control offspring with the LF diet; CHF, control offspring with the HF diet; HLF, prenatal hypoxia offspring with the LF diet; HHF, prenatal hypoxia offspring with the HF diet; PH, prenatal hypoxia. **P < 0.001, two‐way anova. ##*P* < 0.001, Bonferroni post hoc test, comparing between the PH and control offspring receiving the same diet

To investigate the BK channel functions in PE‐induced vasoconstrictions, a selective BK inhibitor iberiotoxin (IbTx) was used. After BK channels were blocked by IbTx (0.1 μmol/L), phenylephrine‐induced vasoconstrictions were significantly enhanced in four groups (Figure 3A). However, the Δ phenylephrine‐induced vasoconstrictions and Δ area under the concentration‐response curve (AUC) after the IbTx treatment was significantly increased in HF‐fed offspring, regardless of exposure to prenatal hypoxia or not (Figure 3A). Whole‐cell K^+^ current density in MASMCs was significantly increased in HF‐fed offspring, IbTx (0.1 μmol/L) markedly decreased the K^+^ currents in four groups (Figure 3B, C and D). BK currents were significantly increased in CHF and HHF groups (Figure 3E).

**Figure 4:**
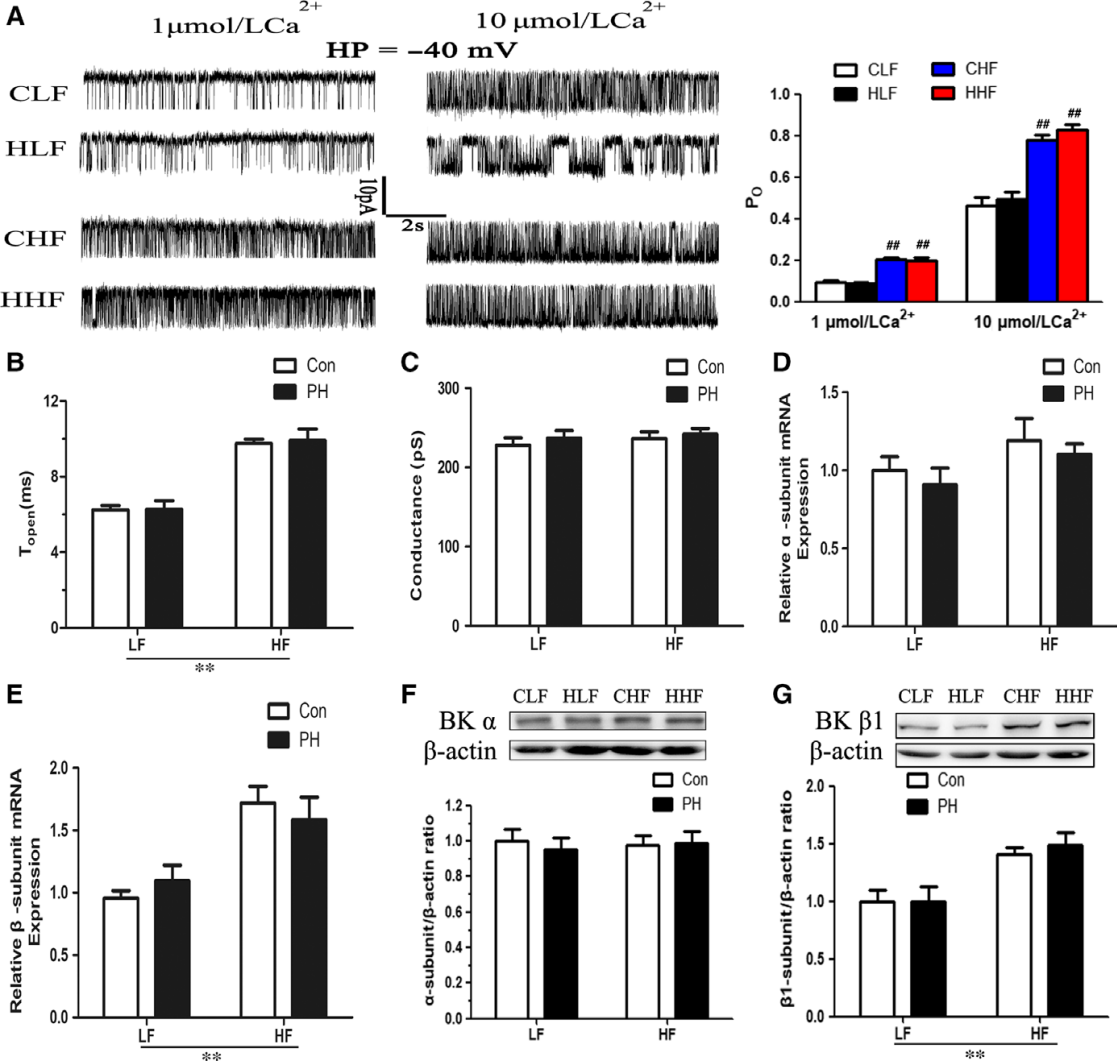
The effect of prenatal hypoxia and postnatal HF diet on properties of single Ca^2+^ sensitivity of large‐conductance calcium‐activated K^+^ (BK) channels. A, Representative single‐channel recordings from inside‐out patches and open probability of BK channels monitored at –40 mV in the presence of 3 and 10 μmol/L free Ca^2+^. B, Dwell time of open state. C, Conductance of BK channels. (D and E) The mRNA expression of BK channel α and β1‐subunits in mesenteric arteries. (F and G) BK channel α (125 kDa) and β1 (22 kDa) subunit proteins in mesenteric arteries (n = 6/group). CLF, control offspring with the LF diet; CHF, control offspring with the HF diet; HLF, prenatal hypoxia offspring with the LF diet; HHF, prenatal hypoxia offspring with the HF diet. PH, prenatal hypoxia. **P < 0.001, two‐way anova. ##*P* < 0.001, Bonferroni post hoc test

The Ca^2+^ sensitivity of BK channels was determined using inside‐out patch‐clamp methodology. In the presence of two physiologically relevant concentrations of intracellular free Ca^2+^ (3 and 10 μmol/L), the single BK channel currents in inside‐out membrane patches from MASMCs of four groups were recorded at –40 mV (Figure 4A). The open probability (Po) and mean open dwell times of BK channels at each Ca^2+^ concentration were increased in the MASMC of HF‐fed offspring, independent to the prenatal hypoxia factor, single‐channel slope conductance was unchanged among the four groups (Figure 4).

To assess whether the increased BK activities in MASMCs of HF‐fed offspring were caused by altered channel molecular constitutions, mRNA and protein expression of BK channel α and β1‐subunits in arteries were determined. The mRNA and protein expression of BK channel α subunit (BK α) were not changed among four groups (Figure 4D, F). However, BK channel β1‐subunit (BK β1) mRNA and protein were significantly increased in HF‐fed offspring independent to prenatal hypoxia factor. No difference in mRNA and protein expression of BK β1 was observed between HLF and CLF (Figure 4E, G).

### 3.5 Effect of prenatal hypoxia and postnatal HF diet on Cav1.2 channel functions and protein expression

**Figure 5:**
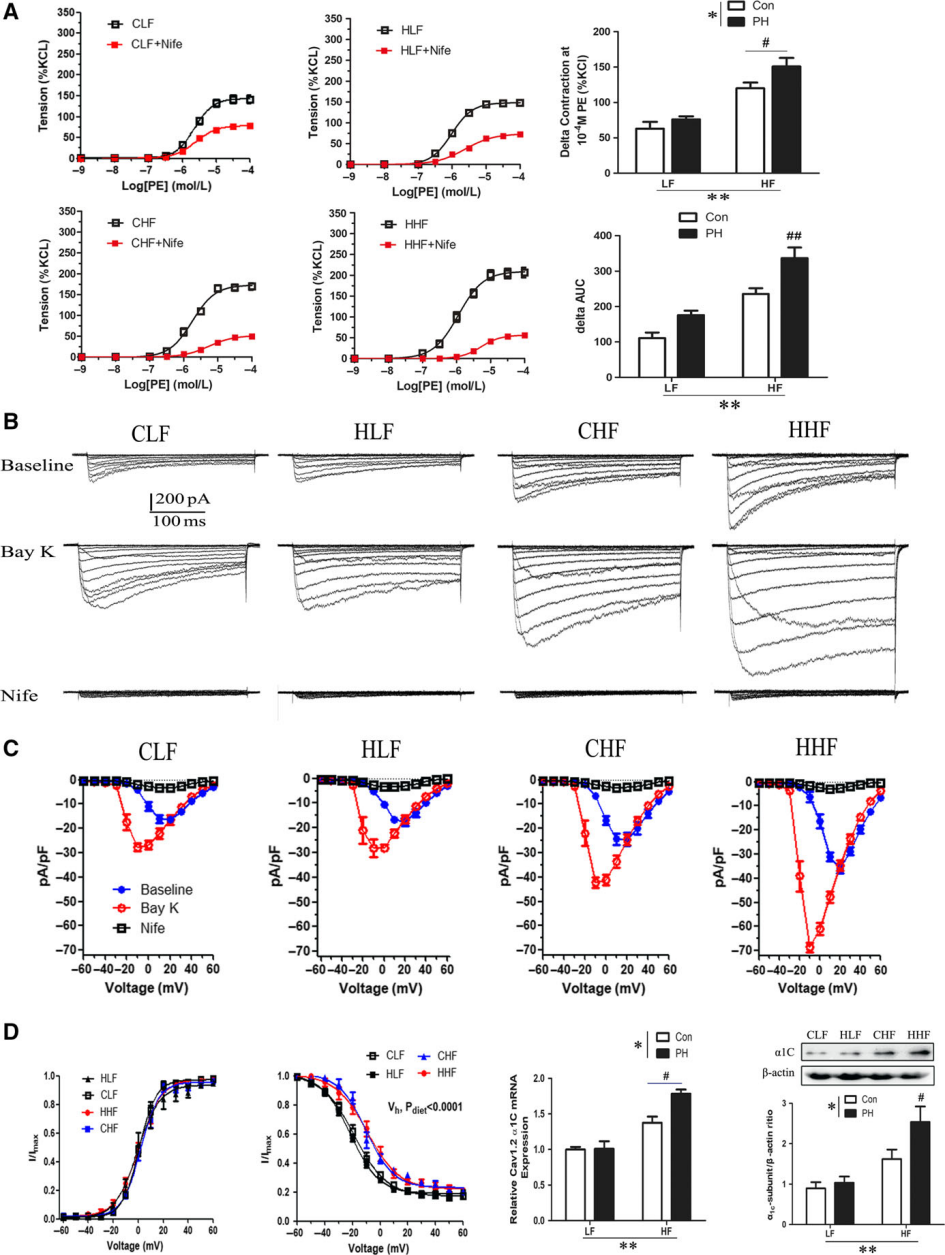
The effect of prenatal hypoxia and postnatal HF diet on L‐type voltage‐gated Ca^2+^ (Cav1.2) channels in mesenteric arteries and myocytes. A, Vascular responses to phenylephrine (PE) in absence or presence of nifedipine (Nife). B, Representative traces of Ca^2+^ channel currents in the absence (Baseline) or presence of Bay K8644 (BayK; 5 μmol/L) or Nife (1 μmol/L). C, I‐V relationships of the Cav1.2 channel currents in mesenteric artery myocytes from CLF, HLF, CHF, and HHF groups. Steady‐state voltage‐dependent activation (D) and inactivation (E) curves of Cav1.2 channel currents in mesenteric myocytes. The mRNA (F) and protein (G) expression of Cav1.2 channel (α1C) subunit (239 kDa) in mesenteric arteries (n = 6 per group). V_h_, Half‐maximal voltages. AUC, area under the concentration‐response curve; CLF, control offspring with the LF diet; CHF, control offspring with the HF diet; HLF, prenatal hypoxia offspring with the LF diet; HHF, prenatal hypoxia offspring with the HF diet. *P < 0.05, **P < 0.001, two‐way anova. #*P* < 0.05, ##*P* < 0.001, Bonferroni post hoc test, comparing between the PH and control offspring receiving the same diet

To determine functional voltage‐dependent Cav1.2 channels, a selective Cav1.2 channel inhibitor nifedipine (Nife) was used in ex vivo vessel ring experiment. In the presence of Nife, phenylephrine‐induced vasoconstrictions were significantly decreased in four groups (Figure 5A). Furthermore, the Δ phenylephrine‐induced vasoconstrictions and Δ AUC following Nife were significantly increased in HF‐fed offspring (Figure 5A). Importantly, a further significant increase of Δ phenylephrine‐induced vasoconstrictions was observed in HHF compared with CHF, while no difference was observed between CLF and HLF (Figure 5A).

Voltage‐dependent Cav1.2 channels were further investigated using conventional whole‐cell patch clamping. Figure 5B shows the representative examples of the whole‐cell Ca^2+^ currents recorded in MASMCs of four groups in the absence and presence of BayK 8644 or Nife. HF diet significantly increased the peak density of the baseline voltage‐dependent inward Ca^2+^ current (at +10 mV) in MASMCs of both control and PH offspring (Figure 5B, Table 2). Furthermore, the peak baseline current in HHF was significantly higher than that in CHF, whereas no difference was found between CLF and HLF (Figure 5B, Table 2). In the presence of BayK 8644 (5 μmol/L), the peak inward current densities were significantly increased and shifted to the left in MASMCs of all groups (Figure 5B, Table 2). The peak current densities following BayK 8644 in HF‐fed offspring were significantly higher than that in LF‐fed offspring. Besides, prenatal hypoxia resulted in a further significant increase of peak currents compared with the control. Nife (1 μmol/L) almost completely inhibited the inward currents in all groups, suggesting the recorded inward currents flew into cells through Cav1.2 channels. There was no difference in the voltage dependence activation among the four groups, while the half‐maximal voltages (V_h_) of inactivation curve were significantly decreased in MASMCs in high‐fat offspring, independent from prenatal hypoxia (Figure 5 D, E and Table 2). RT‐qPCR and Western Blot showed that the mRNA and protein expression of the pore‐forming α1C‐subunit of Cav1.2 channels were significantly increased in HF‐fed offspring, which were exacerbated by prenatal hypoxia.

**Table 2:** Properties of CaV1.2 currents in mesenteric arterial myocyte

	LF diet		HF diet		Two‐way anova			
	Con	PH	Con	PH	PH	Diet		Int
Peak current density (pA/pF)								
Baseline	–16.16 ± 1.44	–17.27 ± 1.29	–24.69 ± 2.46	–36.06 ± 1.74	*		*	*
Bay K 8644	–26.81 ± 1.84	–28.25 ± 3.71	–42.31 ± 2.15	–68.67 ± 2.00†	*		*	*
Nife	–3.34 ± 0.13	–3.41 ± 0.25	–3.32 ± 0.25	–2.86 ± 0.11				
Half‐maximal voltages								
V_h‐act_ (mV)	0.63 ± 0.42	–0.89 ± 1.36	1.77 ± 0.68	0.32 ± 0.70				
V_h‐inact_ (mV)	–18.70 ± 1.23	–21.58 ± 1.21	–10.08 ± 1.05	–9.37 ± 1.46		*		

Con, control; HF, high fat; Int, interaction; LF, low fat; PH, prenatal hypoxia; V_h‐act_, half‐maximal voltages in voltage‐dependent activation; V_h‐inact_, half‐maximal voltages in voltage‐dependent inactivation.

* *P* < 0.05 for the respective sources of variation (PH, diet, or their interaction) using two‐way anova (Con with LF diet, n = 6; PH with LF diet, n = 7; Con with HF diet, n = 9; PH with HF diet, n = 8).

† *P* < 0.05 (Bonferroni post hoc test compared to the control offspring fed with the same diet).

## 4 DISCUSSION

**Figure 6:**
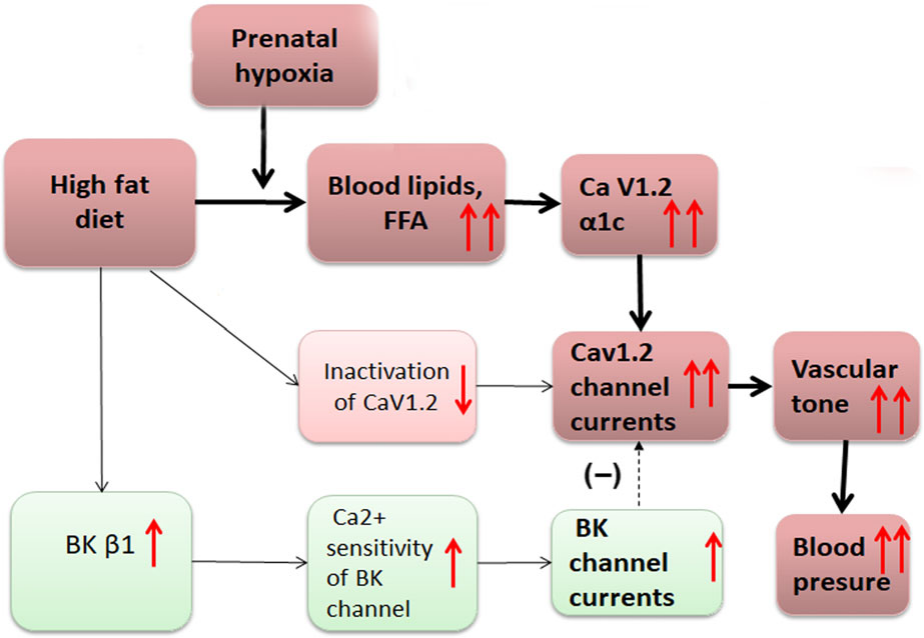
A model for a mechanistic explanation of the effect of prenatal hypoxia and postnatal HF diets on blood pressure (BP) in young adult offspring. Prenatal hypoxia increased the susceptibility to postnatal high‐fat diets, which exacerbated dyslipidemia. Higher plasma FFA may contribute to elevation of Cav1.2 currents in MASMC, increasing vasoconstriction in MA, and resulting in higher BP in HHF group. The up‐regulated β1‐subunits of BK channels contributed to increased BK channel activities in MASMC of rats exposed HF diets as a temporary protective and compensatory measure for the augmented calcium currents that led to the increased vasoconstriction and B

The present study investigated the effects of chronic hypoxia in pregnancy, and determined whether and how this prenatal insult interacted with the postnatal high‐fat diets, affecting vascular functions in young adult offspring. The main findings include: (a) prenatal hypoxia plus postnatal HF diets significantly exacerbated plasma lipid concentrations compared with the HF‐fed only; (b) prenatal hypoxia exacerbated HF‐elevated BP and vascular tone via up‐regulated α1c‐subunits, resulting in increased Cav1.2 channel currents; (c) postnatal HF caused up‐regulated BK β1‐subunits, leading to increased BK channel currents, independent of prenatal hypoxia (Figure 6).

Mounting evidence suggests that IUGR and the following accelerated growth after birth are predictors of adult‐onset diseases, such as hypertension.^19^, ^20^, ^21^ The present study found that the birth weight of rats exposed to chronic prenatal hypoxia was decreased. Through the catch‐up growth, the prenatal hypoxia offspring had comparable weights to controls after 1 month old. Consistent with previous reports,^22^, ^23^ the present study showed that more weight gain was found in the HF‐exposed offspring, which might be due to significantly increased caloric intake. In addition, the offspring with both prenatal hypoxia and postnatal hyper‐caloric HF diets showed much higher triacylglycerol, cholesterol, low‐density lipoprotein‐cholesterol, and free fatty acids in circulation, suggesting that the synergistic effects of prenatal hypoxia and postnatal HF diet were associated with an early onset of dyslipidemia in this model. Notably, only one single factor either prenatal hypoxia or postnatal HF diets affected some indexes of the blood values, while those two factors together could significantly make it worse for plasma triacylglycerol, cholesterol, and free fatty acids. Clinical significance of this finding includes: to those with history of prenatal insults, special attention should be given to avoid high fat in later life.

The present study demonstrated that prenatal hypoxia alone did not significantly change BP in offspring at 4‐month‐old if postnatal diets were normal or healthy. However, the baseline BP was significantly increased in the offspring following feeding high fat for 12 weeks, which was exacerbated by prenatal hypoxia. One previous report suggested that postnatal HF diets did not alter the baseline BP in the rat offspring either born from control dams or dams exposed to hypoxia during late gestation.^24^ The discrepancy may be due to the differences in experimental conditions, such as the duration of hypoxia and feeding of high fat. It has been demonstrated that high‐fat diets could elevate BP in both human and animal models.^25^, ^26^, ^27^ The novel information in our results suggests that the offspring exposed to prenatal hypoxia was more susceptible to hypertension when postnatal high‐fat diet was offered.

It is known that vascular tone in peripheral resistance arteries plays a dominant role in regulating BP, and vascular tone mainly depends on constrictor state of vascular smooth muscle cells.^28^ To determine possible mechanisms involved in the elevated BP in the offspring exposed to prenatal hypoxia and postnatal HF, peripheral resistance vessels were investigated in the present study. Contrary to the results reported by others using the model of hypoxia during late gestation,^23^ we found that chronic foetal hypoxia significantly exacerbated the PE‐mediated MA constriction following postnatal HF. This discrepancy may be due to the differences in hypoxic conditions and experimental protocols used, such as a longer period of hypoxia and treatments of high‐fat diet in the present study. In addition, we also found that foetal hypoxia alone was able to increase the sensitivity of PE‐induced vasoconstrictions as the EC_50_ values (performance as pD2) were significantly shifted to the left in the offspring compared with controls. The endothelium plays an important role in vascular regulations. In blood vessels, acetylcholine (ACh) acts on cholinergic receptors in the endothelium to produce NO that causes vascular relaxation. In the present study, NO mediation of endothelial dependent relaxation was evaluated using ACh. We found that ACh‐mediated dose‐dependent vasodilatation was reduced in HF‐exposed offspring, independent from prenatal hypoxia factor. These results demonstrated that prenatal hypoxia plus postnatal HF could significantly increase vasoconstrictions in MA, contributing to the elevated BP in HHF offspring.

To determine the mechanisms underlying the increased vascular tone, next of our experiments focused on ion channels on smooth muscle cells of resistance arteries. BK channels in VSMC usually serve as a negative feedback mechanism to counteract membrane depolarisation and vasoconstrictions.^29^, ^30^ Numerous studies showed that dysfunction of BK channels contributed to vascular disorders. For example, impaired BK channel functions in arteriolar smooth muscle cells presented in models of insulin resistance,^31^ diabetes,^32^, genetic obesity^33^ and hypertension.^34^ Therefore, our initial ideas proposed that prenatal hypoxia plus postnatal high‐fat diets may result in a down‐regulation of BK channels in MASMC of young adult offspring, as a mechanism for increased BP and PE‐mediated constriction of MA. However, in the present study, an interesting finding revealed a significant increase rather than the anticipated decrease in BK channel activities such as whole‐cell BK current density was observed in the MA of offspring exposed to postnatal HF, regardless of prenatally treated with normoxia or hypoxia. Previous studies also demonstrated that high‐fat diets could increase BK channel activities in middle cerebral arteries,^35^ aorta,^36^ and coronary arteries.^37^ It is well known that activation of BK channels induces vascular cellular membrane hyper‐polarisation,and then counteract vascular constrictions via reducing Cav1.2 channel activity.^38^ Therefore, in the present study, the HF‐increased BK activities may be temporary protective and compensatory measures for the elevated vasoconstrictions and BP.

Vascular BK channels are composed of pore‐forming α‐ and accessory β1‐subunits.^39^, ^40^ It is well known that β1‐subunits regulate BK channel functions by increasing Ca^2+^ sensitivity and open dwell time of α‐subunits.^41^, ^42^ Through single‐channel recording, the present study found that the effectiveness of Ca^2+^ in activating BK channels at physiological Em was increased in CHF and HHF group, without significant difference between those two groups. Moreover, the mean open dwell times of BK channels in MASMC from HF group were longer than that from LF group. RT‐qPCR and Western Blot analysis showed that β1, not α‐subunits, was increased in mesenteric arteries of HF offspring. Taken together, the new date suggested that the up‐regulated expression of β1‐subunits contributed to the increased Ca^2+^ sensitivity of BK channels, and then led to the increased BK activities in MASMC of the rats exposed HF diets.

It is well known that Cav1.2 channels play important roles in maintaining vascular myogenic tone of small arteries and arterioles, as well as blood pressure.^43^ Under optimum conditions, in the VSMCs, the open‐state probability of Cav1.2 channels is controlled at a low level to maintain vascular myogenic tone. However, during the pathogenesis of hypertension, an increased Ca^2+^ influx through Cav1.2 channels may cause the development of an exaggerated vascular tone and increased peripheral vascular resistance. Numerous studies demonstrated that the up‐regulated expression of Cav1.2 α1C subunit as well as a higher density of Cav1.2 channel currents was found in MASMC from spontaneously hypertensive rats during early stage of their life.^44^, ^45^ In the present study, prenatal hypoxia alone did not cause significant changes in Cav1.2 current density and protein expression in MASMC of the offspring. However, an interesting finding was that an increase in whole‐cell Cav1.2 current density was found in the myocytes of postnatal HF offspring, which was exacerbated by prenatal hypoxia. It has been known that the increased Cav1.2 currents can be the result of the elevated expression of functional channels as well as up‐regulated Po and/or conductance of single Cav1.2 channels. Although the single Cav1.2 channel current was not recorded in the present study, we investigated channel kinetics of channels and found that the voltage dependence activation was similar among the four groups, while the high‐fat diet shifted the voltage‐dependent inactivation curve to more positive potentials, independent from prenatal hypoxia. The rightward shift of the steady‐state inactivation curve may be one of the causes for a marked increase of whole‐cell Cav1.2 currents in HF offspring, while it did not contribute to the difference of the Cav1.2 currents between HHF and CHF cells, because the inactivation curve was similar between the two groups. As 1,4‐Dihydro‐2,6‐dimethyl‐5‐nitro‐4‐(2[trifluoromethyl]phenyl)pyridine‐3‐carboxylic acid methyl ester (Bay K8644, a selective activator of Cav1.2 channels) increased the open probability of Cav1.2 channels to ≈ 1, the Cav1.2 currents after the application of Bay K8644 reflected the number of functional channels.^46^ The present study found that Cav1.2 currents were larger in HHF than in CHF in the presence of Bay K8644, suggesting that prenatal hypoxia may exacerbate the whole‐cell Cav1.2 currents in MASMC of offspring exposed to postnatal HF via increasing the number of functional Cav1.2 channels. In addition, RT‐qPCR and Western Blot analysis showed that the mRNA and protein expression of pore‐forming α1C subunits of Cav1.2 channels were increased in MA of offspring exposed high‐fat diet and these alterations were exacerbated by prenatal hypoxia. Although this study did not determine whether other Cav1.2 channel subunits, such as auxiliary α2δ and β subunits, were involved, the new results should warrant further investigation.

Early studies demonstrated that FFAs could increase L‐type Ca^2+^ channel currents in cardiac myocytes, probably through modification of physicochemical properties of the protein or lipid interface.^47^ Plasma higher fatty acids also have been reported to contribute to increased BP via elevation of Ca^2+^ current density in the VSMC.^25^ Uteroplacental insufficiency resulted in alterations in gene expression of hepatic fatty acid‐metabolising enzymes in the foetal, juvenile, and adult male rats, which may contribute to dyslipidemia.^48^ In the present study, elevated plasma free fatty acid was found in postnatal HF offspring, and exacerbated by prenatal hypoxia. Based on the above findings, a model for a mechanistic explanation of abnormal vessel tone and hypertension caused by prenatal hypoxia plus postnatal HF was proposed. Briefly, although prenatal hypoxia alone could not significantly affect the vessel tone and BP in young adult offspring rats, it decreased the physiological reserve as hepatic fatty acid metabolism was altered, which may increase the susceptibility to postnatal high‐fat diets and then result in dyslipidemia (especially the higher FFA) in later life. The increased plasma FFA may contribute to the elevation of Cav1.2 currents in MASMC, increasing vasoconstriction in MA and BP in HHF group. However, more evidence should be obtained through further investigation. In addition, the up‐regulated expression of β1‐subunits of BK channels contributed to increased BK channel activities in MASMC of rats exposed to HF diets, which may be a temporary protective and compensatory measure for the augmented calcium currents that lead to the increased vasoconstriction and BP. We also realize the limitations of the present study. For example, if foetal data under conditions of prenatal hypoxia could be obtained, that will be helpful for further understanding the acute influence on foetal vascular systems following prenatal insults.

In conclusion, the present study demonstrated that the offspring with prenatal hypoxia was more susceptible to hypertension when high‐fat diets were introduced after birth. The new information gained is very important for those children and young adults with history of prenatal hypoxia. Although hypoxia in utero may be unavoidable sometimes, healthy diets after birth may prevent or limit the development of hypertension. This study demonstrated that the up‐regulation of functional Cav1.2 channels in MASMC may be the main cause for the elevated BP in HF‐fed offspring following prenatal hypoxia. Therefore, these results suggest that postnatal interventions, such as avoiding unhealthy factors or targeting Cav1.2 channels, may be effective in reducing risks in cardiovascular diseases in foetal origins.

## CONFLICT OF INTEREST

The authors confirm that there are no conflicts of interest.

## AUTHOR CONTRIBUTIONS

XL, XF, LLu, MS and ZX designed research; XL, XF, LLu, AH, BL, YZ, XR, YL and XC conducted research; XL, XF and LLu analysed date; XL, XF, LLu, MS and ZX wrote the paper and had primary responsibility for final content. All the authors read and approved the final manuscript.
